# Mixed fleet-based two-echelon vehicle routing optimization for cold chain logistics with diverse recharging strategies

**DOI:** 10.1371/journal.pone.0318765

**Published:** 2025-02-13

**Authors:** Yaling Li, Wenzhu Liao, Yuqi Huang

**Affiliations:** Department of Engineering Management, Chongqing University, Chongqing, China; Cardiff's Metropolitan University: Cardiff Metropolitan University, UNITED KINGDOM OF GREAT BRITAIN AND NORTHERN IRELAND

## Abstract

The expansion of cold chain logistics necessitates a substantial fleet of fuel-refrigerated trucks, which presents environmental challenges. Electric vehicles (EVs) offer an environmentally friendly solution for low-carbon development despite the issue of range anxiety. Diverging from the conventional two-echelon distribution structure, this paper explores alternative recharging strategies and introduces an innovative scheme: employing fuel vehicles in suburban areas and EVs in urban central zones. The presented model optimizes economic and environmental considerations to mitigate air pollution and reduce dependence on non-renewable energy sources while providing feasible routes. This study proposes an allocation algorithm and an enhanced ant colony algorithm to address a single-objective two-echelon vehicle routing problem with the mixed fleet (2EVRPMF). The mixed fleet outperforms in terms of both cost and carbon emissions based on numerical experiments. Additionally, the study investigates the influence of battery capacity and recharging rate under various recharge strategies, including their correlation with costs. The findings can provide valuable insights for decision-making in implementing environmentally-friendly logistics within the cold chain industry.

## 1. Introduction

Recently, the rapid growth of e-commerce in fresh food and the improvement of living standards have led to an expansion in cold chain logistics. According to data from the China Federation of Logistics & Purchasing, China’s total demand for cold chain logistics is expected to reach approximately 350 million tons by 2023, reflecting a year-on-year increase of 6.1%. As the cold chain logistics sector continues its steady progression, it has become imperative to expedite the transition towards emission reduction and adopt low-carbon practices for sustainable and healthful development.

With the rapid expansion of the cold chain logistics market, there has been a substantial surge in the utilization of fuel vehicles, leading to significant environmental pollution concerns. In 2022, China witnessed a cumulative emission of pollutants from fuel vehicles amounting to 14.662 million tons, encompassing 7.43 million tons of carbon monoxide (CO), 1.912 million tons of hydrocarbons (HC), 5.267 million tons of nitrogen oxides (NOx), and 53 thousand tons of particulate matter (PM) [1]. Moreover, the release of greenhouse gases from vehicle exhaust exacerbates the greenhouse effect and exerts profound impacts on ecosystems across various regions. The cold chain industry should therefore consider incorporating new energy-powered refrigerated vehicles into their green logistics initiatives.

The existing technical limitations, however, present challenges for electric vehicles (EVs), such as range anxiety and longer recharging times compared to refueling. These factors impede their suitability for long-distance distribution due to inadequate infrastructure for public recharging stations. Consequently, current EVs are unable to fully replace conventional fuel vehicles, necessitating alternative options for logistics enterprises.

The initial approach suggested in prior research is to take into account hybrid vehicles, which capitalize on the combined advantages of both types of vehicles. The second approach involves a hybrid fleet that takes into account both types [2]. Existing articles mainly focus on scenarios involving distribution centers and demand points. The common assumption is that each demand point is exclusively served by a single vehicle from the origin to the destination [3–5]. However, discussions regarding mixed fleets in the two-stage vehicle routing problem are still limited, with few results applicable to cold chain logistics research. For example, Breunig et al. [6] introduced EVs in the second stage but did not impose strict time windows. Wang et al. [7] concentrated on rural green logistics and emphasized optimizing carbon emissions, storage costs, and distribution center location costs.

Taking the aforementioned constraints into consideration, this paper aims to propose a two-echelon distribution scheme that incorporates a mixed fleet for cold chain logistics. Departing from the conventional two-echelon distribution structure, this approach advocates for the integration of electric refrigerated vehicles in urban central areas while retaining fuel-refrigerated vehicles for use in suburban regions. This innovative method can reduce reliance on non-renewable energy sources and minimize air pollutant emissions.

Charging stations serve as an effective solution to mitigate the range anxiety associated with EVs. Presently, certain studies concentrate on examining charging power [8], while others focus on charging strategies, particularly partial charging [9]. The latter approach is employed in this paper to effectively mitigate the various costs incurred by terminal nodes. In the sensitivity analysis, the impact of charging rate is examined, indirectly reflecting the influence of DC and AC charging on strategy implementation.

Section 2 provides a comprehensive review of the existing literature on three key topics. Section 3 presents a two-echelon vehicle routing model based on mixed fleet composition. Section 4 proposes an integrated solution comprising a vehicle allocation algorithm and an ant colony optimization algorithm. Section 5 validates the effectiveness of the model, and Section 5.4 analyzes the sensitivity of battery capacity and recharging rate Finally, Section 6 encompasses the conclusion, limitations, and future directions.

## 2. Literature review

As the proposed distribution scheme in this paper addresses the Two-Echelon Vehicle Routing Problem with Mixed Fleet (2EVRPMF) for cold chain logistics, this comprehensive model encompasses three key research components: the Electric Vehicle Routing Problem, the Two-Echelon Vehicle Routing Problem, and the Vehicle Routing Problem specific to cold chain logistics. A thorough discussion of relevant literature in these domains is provided.

### 2.1. Electric vehicle routing problem

The EVRP aims to determine the optimal route for EVs considering various constraints and operations [10]. Currently, several variations of EVRP have emerged, including incorporating time windows into the routing problem [11], considering energy consumption as a key factor in route optimization [12], and addressing chance-constrained multi-compartment scenarios [13]. As recharging processes typically entail significant time, various recharge strategies have garnered widespread attention. The primary energy supply modes for EVs consist of two types: recharging [14–16] and battery replacement. The former can be broadly categorized into three groups [17]: full charging, linear partial charging, and nonlinear partial charging [18–20]. While EVs offer a cleaner option for sustainable development, their limitations in mileage and electric power have prompted researchers to explore the integration of traditional fuel vehicles and EVs. One way is to assemble a mixed fleet of several vehicle types [21,22]. Another way is hybrid electric vehicles (HEVs) [23,24], consuming both electricity and gasoline.

### 2.2. Two-echelon vehicle routing problem

The early literature on 2EVRP primarily focused on the two-echelon capacitated vehicle routing problem [25–28], with the prevalent use of branch and cut algorithms [29,30]. The literature in recent years has witnessed a growing body of research focusing on diverse transportation options, with particular emphasis on the integration of conventional and contemporary tools. Liu et al. [31] proposed a combination of conventional trucks and automatic delivery robots to optimize transportation costs and reduce emissions. Ding et al. [32] introduced a 2EVRP involving electric and conventional vehicles for transporting common goods. Faiz et al. [33] employed fuel trucks for the first echelon distribution while utilizing drones for the second echelon distribution during post-disaster rescue operations. Further breakthroughs have also been made in heuristic algorithms. Enthoven et al. [34] used an adaptive large neighborhood search heuristic method to solve a 2EVRP with intermediate position options. Anderluh et al. [35] employed a metaheuristic that integrated large neighborhood search into a heuristic cuboid splitting approach. The study conducted by Sherif et al. [36] explored green transportation in the battery industry through the utilization of a simulated annealing algorithm and an exchange neighborhood-solving method. Lehmann and Winkenbach [37] employed exact methods for the first stage, followed by heuristic algorithms for the second stage. Despite the availability of multiple approaches, selecting the most optimal one remains challenging [38].

### 2.3. Vehicle routing problem for cold chain logistics

In the context of cold chain logistics for the vehicle routing problem (VRP), sustainability discussions primarily revolve around establishing indicators such as carbon emissions, carbon trading and carbon taxes as cost targets [39–41]. Additionally, comprehensive assessments encompassing social, economic and environmental aspects are conducted [42]. Research on two-echelon distribution in cold chain logistics has been relatively limited. Govindan et al. [43] introduced a 2EVRP model incorporating time windows for the perishable food supply chain and successfully addressed it using a hybrid metaheuristic algorithm. Wang et al. [44] examined a two-echelon logistics framework involving small vehicles and semi-trailer trucks, highlighting the cost advantages of an enhanced ant colony optimization algorithm. Yan et al. [45] conducted a comparative analysis of solutions for different objectives in a location-routing problem. Al Theeb et al. [46] discussed the 2EVRP of vaccines, where vehicles were divided into large and small sizes. While these studies considered a heterogeneous fleet, vehicles in each echelon mainly differed in capacity, rarely in energy type. The literature has examined the single-echelon path problem, which involves a mixed fleet of electric and conventional vehicles (with only one type per service [3,5,47]). In terms of the 2EVRP for cold chain logistics, Zhang et al. [48] proposed a simplified two-stage hybrid transportation scenario that does not incorporate charging strategy. The summary of previous review studies is given in Table 1.

**Table 1:** Summary of previous review studies.

Researchers’ name	Type of fleet	Type of fuel used		Product type	Charging Strategy	solving method
		electricity	Combustion			
Enthoven et al. [34]	HE	√	√	ordinary	×	ALNS
Liu et al. [41]	HO		√	perishables	×	SAA
Anderluh et al. [35]	HE	√	√	ordinary	×	ALNS
Chen et al. [39]	HO		√	perishables	×	HSATA
Sherif et al. [36]	HE		√	ordinary	×	SA
Liu et al. [31]	HE	√	√	ordinary	×	C-AIA
Al Theeb et al. [46]	HE		√	perishables	×	GRS
Chen et al. [3]	HE	√	√	perishables	×	VNS
Ding et al. [32]	HE	√	√	ordinary	FR,PR	IDS
Hou et al. [40]	HE		√	perishables	×	GA
Faiz et al. [33]	HE	√	√	ordinary	×	CG
Lehmann and Winkenbach [37]	HO	√		ordinary	×	EX-ALNS
Ma et al. [47]	HE	√	√	ordinary	×	ALNS
Zhang et al. [48]	HE	√	√	perishables	×	ACO
This paper	HE	√	√	perishables	FR, PR	ACO

HE: Heterogeneous, HO: Homogenous, HSATA: hybrid simulated annealing and tempering algorithm, SAA: Simulated Annealing Algorithm, C-AIA: clustering-based artificial immune algorithm, GRS: greedy random search, VNS: Variable Neighborhood Search, IDS: intelligent dispatch scheme, GA: genetic algorithm, CG: column generation, ALNS: adaptive large neighborhood search, EX: exact formulation, ACO: ant colony optimization.

As discussed above, owing to current technical limitations, EVs still face challenges such as mileage anxiety and longer recharging times compared to refueling. These factors make them less suitable for long-distance distribution. Limited attention has been given to the integration of a mixed fleet of fuel vehicles and EVs. Additionally, the exploration of the partial recharge strategy remains scarce in the context of 2EVRP [49]. Therefore, this paper aims to introduce a novel concept: the 2EVRP with a Mixed Fleet (2EVRPMF) for cold chain logistics. The distinctive features of this research include:

1. The use of fuel vehicles and EVs in the first and second echelons, respectively, forming a mixed fleet.
2. The adoption of different recharge strategies, namely partial recharge and full recharge, in the 2EVRPMF. An algorithm for selecting a recharging upper limit is developed specifically for the partial recharge strategy.
3. The introduction of a customized and enhanced Ant Colony Optimization method for route planning within the 2EVRPMF.

## 3. Model construction

### 3.1. Problem description

**Fig 1:**
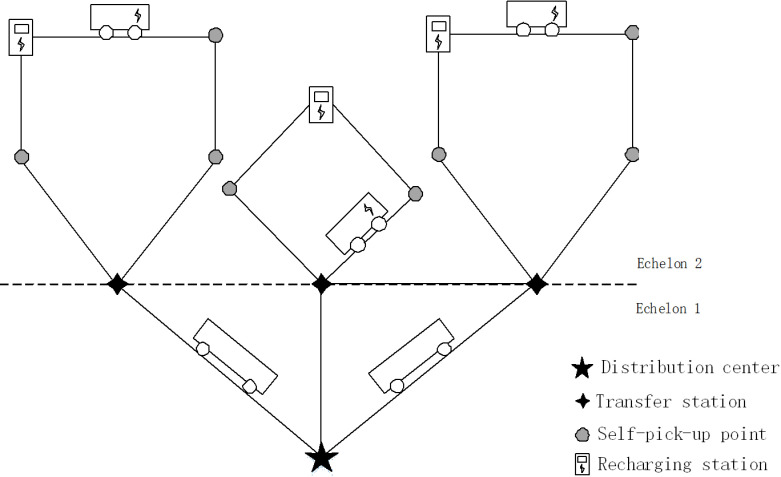
Two-echelon distribution network. The definition of sets, parameters, and variables of this 2EVRPMF is shown in Table 2.

The paper introduces a two-echelon distribution network. The primary echelon comprises a solitary distribution center and multiple transfer stations (satellites). These stations are serviced by a fleet of fuel vehicles. In the secondary echelon, there are additional transfer stations, self-pick-up points (customers), and recharging stations. The total number of EVs is fixed, although the specific allocation for each EV is not predetermined. Following the policy that establishes a 1:1 ratio between the number of new public charging stations and newly promoted EVs in the public sector [50], EVs have the option to visit nearby recharging stations if their charge level becomes low (as depicted in Fig 1). The coordinates of the distribution center, transfer stations, self-pick-up points, and recharging stations are provided. The demand for self-pick-up points is specified. The demand for transfer stations is calculated as the sum of the demands for the served self-pick-up points. Several assumptions are made to facilitate the study, including:

**Table 2:** Mathematical notations.

Sets	
$V_{0}=\left\{0\right\}$	Distribution center
$V_{S}$	The set of transfer stations
$V_{C}$	The set of self-pick-up points
$V_{R}$	The set of recharging stations
$V_{R}^{'}$	The set of dummy copies of recharging stations
$V_{1}=V_{0}\cup V_{S}$	The set of distribution centers and transfer stations (the nodes of the first echelon)
$V_{2}^{'}=V_{S}\cup V_{C}\cup V_{R}^{'}$	The set of transfer stations, self-pick-up points, and dummy copies of recharging stations (the nodes of the second echelon)
*F*	The set of fuel vehicles
*E*	The set of EVs
*Parameters*	
$c_{fix1}$	Fixed cost of each fuel vehicle
$c_{fix2}$	Fixed cost of each electric vehicle
$c_{f}$	Fuel price per unit
$c_{e}$	Electricity price per unit
$c_{p}$	The unit price of the products
$c_{c}$	The unit price of carbon emission
$r_{1}$	Fuel consumption of transportation per mile
$r_{2}$	Electricity consumption of transportation per mile
$r_{3}$	Electricity consumption of refrigeration per hour
$E_{f}$	The amount of carbon emission of unit fuel
$E_{e}$	The amount of carbon emission of unit electricity
$d_{ij}$	Distance between node *i* and node *j*, $i,j\in V_{1}\cup V_{2}^{'}$
$v_{1}$	Fuel vehicle speed
$v_{2}$	EV speed
$I_{i}$	Recharging rate of station *i*, $i\in V_{R}^{'}$
∂	The spoilage rate of the product
$d_{j}$	Demands of self-pick-up point *j*, $j\in V_{C}$
$m_{1}$	Maximum quantity of fuel vehicles
$m_{2}$	Maximum quantity of EVs
$Q_{1}$	Maximum load of fuel vehicle
$Q_{2}$	Maximum load of EV
*B*	EV battery capacity
*Variables*	
$T_{jf}$	The arrival time of vehicle *f* at node *j* in echelon 1
$t_{j}$	The arrival time of vehicle at node *j* in echelon 2
$B_{Uie}$	The recharging upper limit of EV *e* at recharging station *i*
$R_{ie}$	The recharge amount of EV *e* at recharging station *i,* $e\in E,i\in V_{R}^{'}$
$b_{ie}^{1}$	State of Charge (SoC) of EV *e* at arrival at node *i*, $e\in E,i\in V_{2}^{'}$
$b_{ie}^{2}$	SoC of EV *e* at departure from node *i*, $e\in E,i\in V_{2}^{'}$
$D_{k}$	Quantity of goods satellite *k* needs to transfer to customers, $k\in V_{S}$
$D_{k}^{f}$	Quantity of goods transported by vehicle *f* to satellite *k*, $f\in F,k\in V_{S}$
$Q_{ij}^{1}$	Flow passing through the first echelon arc (*i,j*), $i,j\in V_{1}$
$Q_{ijk}^{2}$	Flow passing through the second echelon arc (*i,j*) and coming from satellite *k,* $i,j\in V_{2}^{'}$
$x_{ijf}$	1 if the vehicle *f* travels from note *i* to *j*, 0 otherwise
$y_{ije}$	1 if the vehicle *e* travels from note *i* to *j*, 0 otherwise
$z_{ie}$	1 if the vehicle *e* starts from transfer station *i*, 0 otherwise
$w_{e}^{k}$	1 if the vehicle *e* is used by satellite *k*, 0 otherwise
$z_{kj}$	1 if customer/recharging station *j* is served by satellite *k*, 0 otherwise

(1) The distribution center is well-stocked with sufficient goods.
(2) The demand of transfer stations is divisible, allowing goods at a transfer station to be delivered by multiple vehicles if necessary.
(3) The demand of self-pick-up points is indivisible, meaning each self-pick-up point can only be served by one vehicle at a time.
(4) Fuel and battery discharge are independent of load and solely depend on the distance traveled.
(5) Each transfer station is equipped with a recharging station, and EVs leave the transfer station with a 100% State of Charge (SoC).
(6) Service times and time windows at the self-pick-up points are not considered in the model.

### 3.2. The optimization model

The objective of the mathematical model for 2EVRPMF is to minimize the overall cost, encompassing fixed costs, energy consumption costs, deterioration costs and carbon emission costs.

#### 3.2.1. Fixed cost.

The fixed cost of a vehicle encompasses expenditures for depreciation, driver remuneration and other associated outlays. This can be expressed as


$$C_{1}=c_{fix1}\underset{i\in V_{0}}{\sum }\underset{j\in V_{1}}{\sum }\underset{f\in F}{\sum }x_{ijf}+c_{fix2}\underset{i\in V_{s}}{\sum }\underset{j\in V_{2}^{'}}{\sum }\underset{e\in E}{\sum }y_{ije}$$ (1)

#### 3.2.2. Energy consumption cost.

The energy consumption is derived from both the transportation and refrigeration processes, encompassing three distinct components: fuel consumption in the primary echelon, electricity consumption in the secondary echelon and electricity consumption for refrigeration at each echelon. The cost associated with energy consumption can be expressed as


$$C_{2}=C_{21}+C_{22}+C_{23}$$

$$=c_{f}r_{1}\underset{i\in V_{1}}{\sum }\underset{j\in V_{1}}{\sum }\underset{f\in F}{\sum }x_{ijf}d_{ij}+c_{e}r_{2}\underset{i\in V_{2}^{'}}{\sum }\underset{j\in V_{2}^{'}}{\sum }\underset{e\in E}{\sum }y_{ije}d_{ij}+c_{e}r_{3}\left(\frac{\sum_{i\in V_{1}}\sum_{j\in V_{1}}\sum_{f\in F}x_{ijf}d_{ij}}{V_{1}}+\frac{\sum_{i\in V_{2}^{'}}\sum_{j\in V_{2}^{'}}\sum_{e\in E}y_{ije}d_{ij}}{V_{2}}+\underset{e\in E}{\sum }\underset{i\in V_{R}^{'}}{\sum }\frac{R_{ie}}{I_{i}}\right)$$ (2)

#### 3.2.3. Deterioration cost.

Deterioration of perishable goods occurs during both the transportation and unloading processes. For simplification purposes, it is assumed that the service time at the customer node is zero. Therefore, the cost of deterioration is considered as damage incurred during transportation.

The arrival time at transfer stations in the first echelon is influenced by both the serving sequence and assigned vehicles. This phenomenon can be mathematically represented as follows


$$T_{jf}=0，\forall f\in F,\forall j\in V_{0}$$ (3)

$$T_{jf}=\underset{i\in V_{1}}{\sum }x_{ijf}\left(T_{if}+\frac{d_{ij}}{V_{1}}\right),\forall f\in F,\forall j\in V_{S},i\neq j$$ (4)

The arrival time at each node in the second echelon is considered under two scenarios when EVs are utilized, presented as

1) the nlformer node is a customer.


$$t_{j}=\underset{e\in E}{\sum }\underset{i\in V_{C}\cup V_{S},i\neq j}{\sum }y_{ije}\left(t_{i}+\frac{d_{ij}}{V_{2}}\right),\forall j\in \left(V_{2}^{'}-V_{S}\right)$$ (5)

2) the former node is a recharging station


$$t_{j}=\underset{e\in E}{\sum }\underset{i\in V_{R}^{'},i\neq j}{\sum }y_{ije}\left(t_{i}+\frac{d_{ij}}{V_{2}}+\frac{R_{ie}}{I_{i}}\right),\forall j\in \left(V_{2}^{'}-V_{S}\right)$$ (6)

According to the literature [51,52], a commonly utilized function is introduced for assessing the quality of refrigerated goods, given as


$$D\left(t\right)=D_{0}e^{-\partial t}$$ (7)

To specify the amount of goods delivered by fuel vehicles in the first echelon, $D_{j}^{f}$ is introduced as it is related to the deterioration cost.


$$C_{3}=C_{31}+C_{32}=\underset{f\in F}{\sum }\underset{j\in V_{S}}{\sum }c_{p}D_{j}^{f}\left(1-e^{-\partial T_{jf}}\right)+\underset{j\in V_{C}}{\sum }c_{p}d_{j}\left(1-e^{-\partial t_{j}}\right)$$ (8)

#### 3.2.4. Carbon emission cost.

The combustion of fuel oil and the generation of electricity both result in direct or indirect carbon emissions. In light of the current government-led carbon trading system, it is imperative to establish a unit price for carbon emissions. Consequently, this unit price serves as an effective means to curb carbon emissions, presented as


$$C_{4}=C_{41}+C_{42}=c_{c}E_{f}r_{1}\underset{i\in V_{1}}{\sum }\underset{j\in V_{1}}{\sum }\underset{f\in F}{\sum }x_{ijf}d_{ij}+c_{c}E_{e}r_{2}\underset{i\in V_{2}^{'}}{\sum }\underset{j\in V_{2}^{'}}{\sum }\underset{e\in E}{\sum }y_{ije}d_{ij}$$ (9)

The objective is to propose an optimization model for 2EVRPMF, to minimize the overall cost including fixed costs, energy consumption costs, deterioration costs, and carbon emission costs, given as.


$$\min C=C_{1}+C_{2}+C_{3}+C_{4}$$ (10)

s.t.


$$\underset{f\in F}{\sum }\underset{j\in V_{S}}{\sum }x_{ijf}\leq m_{1},\forall i\in V_{0}$$ (11)

$$\underset{e\in E}{\sum }\underset{j\in V_{C}\cup V_{R}^{'}}{\sum }y_{ije}\leq m_{2},\forall i\in V_{S}$$ (12)

$$\underset{i\in V_{1},i\neq j}{\sum }x_{ijf}=\underset{k\in V_{1},k\neq j}{\sum }x_{jkf},\forall j\in V_{1},\forall f\in F$$ (13)

$$\underset{i\in V_{2}^{'},i\neq j}{\sum }y_{ije}=\underset{k\in V_{2}^{'},i\neq j}{\sum }y_{jke},\forall j\in V_{2}^{'},\forall e\in E$$ (14)

$$\underset{i\in V_{1}}{\sum }x_{ijf}\leq 1,\forall j\in V_{S},\forall f\in F$$ (15)

$$\underset{e\in E}{\sum }\underset{i\in V_{2}^{'}}{\sum }y_{ije}=1,\forall j\in V_{C}\cup V_{R}^{'},i\neq j$$ (16)

$$\underset{e\in E}{\sum }\underset{i\in V_{S}}{\sum }\underset{j\in V_{R}^{'}}{\sum }y_{ije}=0$$ (17)

$$\underset{e\in E}{\sum }\underset{i\in V_{R}^{'}}{\sum }\underset{j\in V_{R}^{'}}{\sum }y_{ije}=0$$ (18)

$$D_{k}=\underset{j\in V_{C}}{\sum }z_{kj}d_{j},\forall k\in V_{S}$$ (19)

$$\underset{f\in F}{\sum }D_{j}^{f}=D_{j},\forall j\in V_{S}$$ (20)

$$0\leq D_{j}^{f}\leq Q_{1}$$ (21)

$$\underset{i\in V_{1},i\neq j}{\sum }Q_{ij}^{1}-\underset{i\in V_{1},i\neq j}{\sum }Q_{ji}^{1}= \{\begin{array}{c} D_{j},jisnotthedepot\\ -\underset{j\in V_{S}}{\sum }D_{j},otherwise \end{array},\forall j\in V_{1}$$ (22)

$$\underset{i\in V_{2}^{'},i\neq j}{\sum }Q_{ijk}^{2}-\underset{i\in V_{2}^{'},i\neq j}{\sum }Q_{jik}^{2}= \{\begin{array}{c} z_{kj}d_{j},jisnotasatellite\\ -D_{j},otherwise \end{array},\forall j\in V_{2},\forall k\in V_{S}$$ (23)

$$Q_{ij}^{1}\leq Q_{1}\underset{f\in F}{\sum }x_{ijf},\forall i,j\in V_{1},i\neq j$$ (24)

$$Q_{ijk}^{2}\leq Q_{2}\underset{e\in E}{\sum }y_{ije}z_{kj},\forall i\in V_{2}^{'},\forall j\in V_{C}\cup V_{R}^{'},i\neq j,\forall k\in V_{S}$$ (25)

$$\underset{i\in V_{S}}{\sum }Q_{ij}^{1}=0,\forall j\in V_{0}$$ (26)

$$\underset{i\in V_{C}\cup V_{R}^{'}}{\sum }Q_{ijk}^{2}=0,\forall j\in V_{S}$$ (27)

$$b_{ie}^{2}=B,\forall i\in V_{S},e\in E$$ (28)

$$b_{ie}^{2}=B_{Uie},\forall i\in V_{R}^{'},e\in E$$ (29)

$$b_{ie}^{2}=b_{ie}^{1},\forall i\in V_{C},e\in E$$ (30)

$$b_{je}^{1}\leq b_{ie}^{2}-r_{2}d_{ij}y_{ije}+B\left(1-y_{ije}\right),\forall i,j\in V_{2}^{'},i\neq j,\forall e\in E$$ (31)

$$b_{ie}^{1}\geq 0.2B,\forall i\in V_{C}$$

∀e∈E (32)

$$R_{ie}=B_{Uie}-b_{ie}^{1}，\forall i\in V_{R}^{'},\forall e\in E$$ (33)

$$z_{jl}\leq \underset{f\in F}{\sum }\underset{i\in V_{1}}{\sum }x_{ijf},\forall j\in V_{S},l\in \left(V_{C}\cup V_{R}^{'}\right)$$ (34)

$$\left(\underset{i\in V_{2}^{'},i\neq j}{\sum }y_{ije}\right)w_{e}^{k}=z_{kj},\forall j\in V_{C}\cup V_{R}^{'},\forall e\in E,\forall k\in V_{S}$$ (35)

$$\{\begin{array}{c} 0\leq B_{Uie}\leq B,\forall i\in V_{R}^{'},e\in E,whenPR\\ B_{Uie}=B,\forall i\in V_{R}^{'},e\in E,whenFR \end{array}$$ (36)

$$x_{ijf}\in \left\{0,1\right\},\forall i,j\in V_{1},\forall f\in F$$ (37)

$$y_{ije}\in \left\{0,1\right\},\forall i,j\in V_{2}^{'},\forall e\in E$$ (38)

$$z_{kj}\in \left\{0,1\right\},\forall k\in V_{S},\forall j\in V_{C}\cup V_{R}^{'}$$ (39)

$$w_{e}^{k}\in \left\{0,1\right\},\forall k\in V_{S},\forall e\in E$$ (40)

$$Q_{ij}^{1}\geq 0,\forall i,j\in V_{1}$$ (41)

$$Q_{ijk}^{2}\geq 0,\forall i,j\in V_{2}^{'}$$ (42)

Eqs. (11) and (12) ensure that the number of vehicles utilized in each echelon does not exceed the total available vehicle count. Eqs. (13) and (14) guarantee a balanced outflow for all nodes within each echelon. Eq. (15)–(18) represent the regulations governing each echelon. Eq. (15) stipulates that one transfer station is visited by vehicles from the first echelon, with each vehicle serving it no more than once. Eq. (16) indicates that one customer or recharging station is visited by vehicles from the second echelon and served only once. Eq. (17) implies that it is not permissible to directly visit a recharging station from transfer stations. Eq. (18) indicates that consecutive visits to two recharging stations are prohibited. Eq. (19) signifies that the quantity of goods required to be transferred by each transfer station equals the total demand of customers to be served. Eq. (20) elucidates that the amount of goods needed to be transferred by each transfer station is determined by the vehicles in the first echelon. Eq. (21) elucidates that the amount of goods needed to be transferred by each transfer station is determined by the vehicles in the first echelon. Eqs. (22) and (23) demonstrate the balanced outflow of each node, excluding the depot where the outflow equals the total demand of transfer stations. Additionally, for second echelon transfer stations, the outflow corresponds to their assigned unknown demand. Eqs. (22) and (23) also serve to prevent sub tours. The capacity constraints are formulated in Eqs. (24) and (25) respectively. Eqs. (26) and (27) indicate vehicles returning to depots or transfer stations without any load. Different states of charge (SoC) of battery upon leaving a node are illustrated in Eqs. (28)–(30): when departing from transfer stations, the SoC is full; when departing from recharging stations, it is limited by EVs’ recharging limit. For customer nodes, the SoC status remains unchanged from when the vehicle arrives. Eq. (31) represents the constraint on electricity consumption. Eq. (32) indicates that the safe SoC for EVs upon reaching a customer is 0.2B. Eq. (33) calculates the amount of recharge required for an EV at a recharging station. Eq. (34) states that the transfer station can only serve nodes in the second echelon if it is served by vehicles from the first echelon. Furthermore, Eq. (35) ensures that transfer station *k* serve for customer or recharging station *j* only when vehicle *e* serves *j* and is used by *k*. Eq. (36) presents the range of recharging limits for two different recharge strategies: partial recharge and full recharge. Finally, Eqs. (37)–(42) specify the domain of variables.

## 4. Model solution

The demand for transfer stations in the 2EVRPMF is determined by the overall customer demand. Thus a bottom-up approach is employed to address both the MDEVRP in echelon 2 and the split-delivery vehicle routing problem in echelon 1.

### 4.1. Vehicle allocation

The proposed paper presents a vehicle allocation algorithm based on distance to determine the number of EVs assigned to each transfer station, thereby dividing the MDEVRP problem. The fundamental concept is outlined as follows:

1) The demand of each transfer station is calculated. The demand for each transfer station is calculated using $n=\left\lfloor d/c_{e}\right\rfloor$, where *d* represents the demand and $c_{e}$ represents the capacity of EVs. Here, *n* denotes the number of EVs allocated in the first-round plan.
2) Any remaining unsatisfied demand is determined. A loop statement is employed to compare and incrementally assign one additional EV to the transfer station with the highest remaining demand. This iterative process persists until all EVs are assigned or there are no unfulfilled demands left.

### 4.2. Distribution route selection

Due to inherent characteristics of positive feedback, heuristic search, and enhanced solving efficiency, Ant colony optimization algorithm (ACO) proves to be an invaluable tool in addressing 2EVRP [53,54]. Consequently, this present study employs ACO as a methodology for determining distribution paths in two echelons.

#### 4.2.1. Transfer probability.

The transfer probability serves as a crucial determinant for ants in selecting the next node. which is established based on the pheromone levels and heuristic function of the path connecting the previous node and the candidate node, depicted as


$$p\_value= \{\begin{array}{c} \frac{Tau^{\alpha }*Eta^{\beta }}{\sum_{s\in allow_{k}}Tau^{\alpha }*Eta^{\beta }},s\in allow_{k}\\ 0,s\notin allow_{k} \end{array}$$ (43)

where Tau is pheromone. Initially, each path has a pheromone value of 1. This value is subsequently updated through the cumulative selection of ants and the volatilization process. The heuristic function $Eta=1/dist_{ij}$ represents the expectation of ant transfer from node *i* to node *j*. A smaller distance implies a higher expectation. The weight coefficients *α* and *β* determine the influence of pheromone and heuristic function respectively. Finally, $allow_{k}$ denotes a collection of nodes that an ant *k* needs to visit.

#### 4.2.2. Pheromone update.

The pheromone release by each ant occurs along the path it traverses. Over time, the accumulation of pheromone concentration on the optimal route gradually intensifies. Consequently, upon completion of a full cycle by all ants, the total sum of released pheromone concentrations $\Delta \tau_{ij}$ on each path can be determined.


$$\Delta \tau_{ij}=\sum_{k=1}^{n}\Delta \tau_{ij}^{k}$$ (44)

$$\Delta \tau_{ij}^{k}= \{\begin{array}{c} Q/L_{k},\text{ant }k\text{travels from node }i\text{to node }j\\ 0,\text{other} \end{array}$$ (45)

where $\Delta \tau_{ij}^{k}$ represents the concentration of pheromones released by ant *k* on the path between node *i* and node *j*. When ant *k* travels from node i to node *j*, this concentration is calculated as the total amount of pheromones *Q* released by the ant in a cycle divided by the length of all possible paths taken, with any remaining value equal to 0.

The pheromone concentration in the original path gradually diminishes over time, while simultaneously, all ants complete a cycle. Consequently, the new generation exhibits a reduced level of pheromone concentration, presented as


$$\tau_{ij}\left(t+1\right)=\left(1-\rho \right)\tau_{ij}\left(t\right)+\Delta \tau_{ij}$$ (46)

where *ρ* represents the factor of pheromone volatilization, and *t* denotes the number of generations.

#### 4.2.3. Point selection.

The transfer rule controlling parameter $r_{0}$ is introduced to prevent early convergence if the node with the highest transition probability is selected. A random number *r* within the range of [0,1] is generated. If $r\leq r_{0}$, the next node is chosen based on its transfer probability; otherwise, the roulette method is used for selecting the next node.

The Roulette selection strategy is based on the transfer probability proportion, where the probability of selecting a node $\frac{p_{valuei}}{\sum_{i\in V_{2}^{'}}p_{value}}$ determines its likelihood of being chosen. Consequently, even suboptimal solutions have a certain chance of being selected, thereby preventing convergence to local optima.

#### 4.2.4. Measures improvement.

In order to enhance the global convergence of ACO, this paper introduces a genetic mutation process and a pheromone rollback mechanism. The genetic mutation process is executed at an end of each iteration [55], where two nodes from the best routes found in that iteration are exchanged to achieve better results. The number of mutations is correlated with the frequency of pheromone rollbacks, resulting in faster problem-solving speed. Additionally, the pheromone rollback mechanism occurs when consecutive iterations yield identical optimal routes. It rolls back the pheromone levels of these routes to their earliest occurrence while simultaneously adjusting the volatilization factor. This improvement can be expressed as


$$\rho = \{\begin{array}{c} \rho_{0}+c*\rho_{add},\rho \leq \rho_{max}\\ \rho_{max},\rho >\rho_{max} \end{array}$$ (47)

$$\tau_{ij}\left(t+n\right)=\left(1-\rho \right)\tau_{ij}\left(t\right)+\rho \Delta \tau_{ij}\left(t\right)$$ (48)

where *c* represents the count of roll back processes, $\rho_{add}$ denotes the increment of volatilization factor, $\rho_{max}$ signifies the maximum value of volatilization factor, and *n* indicates the number of consecutive iterations that remain unchanged.

### 4.3. Recharging limit determination

The determination of a recharging limit method should be investigated when implementing the partial recharge (PR) strategy. To simplify this determination process, a candidate matrix for the recharging limit is set as $\text{Bu=}\left[0.6,0.7,0.8,0.9,1\right]\text{*B}$ within an optional range. The steps for this determination process are outlined as follows:

1. establish a pheromone matrix that defines a recharge limit, records the pheromone trail of ants from a customer depot to a specific recharging station and ensures recharging up to a predetermined upper threshold;
2. employ the recharging limit pheromone matrix and the roulette method to determine the specific recharging station i and its corresponding upper limit $B_{Uie}$ when approaching a recharging point;
3. determine the SoC of the EVs upon arrival at station *i*, and calculate the required amount of electricity ΔQ for recharging;
4. if ΔQ is negative, continue with step 2)-3) until ΔQ becomes positive;
5. the recharging limit pheromone matrix is updated after each iteration by incorporating the route of each ant that generates a feasible solution.

## 5. Numerical experiment

### 5.1. Benchmark instances

The benchmark sets utilized in this experiments are derived from the 2EVRP instances proposed by Perboli et al. [27], Hemmelmayr et al. [56] and Baldacci et al. [57] To accommodate the distinctive characteristics of EVs employed as city freighters, including the distribution and quantity of recharging stations, as well as the nominal battery capacity, this study adopts the approach suggested by Schneider et al. [58] and Breunig et al. [6] in EVRP. Specifically, the number of charging stations is determined by rounding one-fifth of the total customer count. The placement of recharging stations prioritizes transfer stations with recharging capabilities. Any remaining stations are then randomly distributed within a range that does not exceed the locations of customers. The nominal battery capacity is determined as B=3.5D, where *D* represents the maximum distance between customers and their nearest recharging station.

The paper investigates the establishment of a self-established logistics network by company A in Beijing [59]. The determination of carbon price is based on data sourced from China’s official carbon trading website [60], combined with an evaluation of carbon emissions per unit energy consumption across various energy sources [61]. Furthermore, the parameters for fuel vehicles in the first echelon and EVs in the second echelon are defined based on commonly employed types of cold chain distribution vehicles in China. The parameters valued are given in Table 3.

**Table 3:** Parameters valued.

Parameter	Value	Parameter	Value
$m_{1}$	3	$Q_{2}$	1200
$m_{2}$	11	*B*	32 kWh
$Q_{1}$	5000	$r_{2}$	0.4 kWh/km
$c_{fix1}$	200 CNY	$r_{3}$	2.5 kWh/h
$c_{fix2}$	200 CNY	$E_{f}$	3.31 kg/kg
$c_{f}$	8 CNY/kg	$E_{e}$	0.95 kg/kWh
$c_{e}$	1 CNY/kWh	$V_{1}$	80 km/h
$c_{p}$	5 CNY each	$V_{2}$	40 km/h
$c_{c}$	87.5 CNY/t	$I_{i}$	4 kWh/min
$r_{1}$	0.1 kg/km	∂	0.002

### 5.2. Parameter tuning

The results can be significantly influenced by the parameters of the heuristic algorithm. Therefore, in line with the tuning approach employed by Keskin and Catay [62] as well as Jie et al. [63], this paper selects three instances from benchmark sets with varying sizes (Set2a_E-n22-k4-s6-17, Set2c_E-n51-k5-s6-12-32-37, Set5_100-5-1b) for parameter optimization. The experiment is conducted ten times for each instance. The average percentage deviation from the optimal solution is then calculated across all solutions for each parameter. Subsequently, adjustments are made to minimize this average percentage deviation of the parameter. This iterative process continues until calibration is completed for all parameters. The initial values of each parameter were determined based on previous literature [64].

The parameter tuning results are presented in Table 4. It is important to note that the weight coefficients of pheromone and heuristic function, denoted as α and β respectively, are adjusted simultaneously as a pair.

**Table 4:** Final values of parameter.

description	notation	value
The amount of ant of each iteration	m	40
weight coefficient of pheromone and heuristic function	α,β	1,7
pheromone volatilization factor	ρ	0.1
the total pheromone released by the ant in a cycle	Q	1
transfer rule controlling parameter	r0	0.5

### 5.3. Results discussion

**Fig 2:**
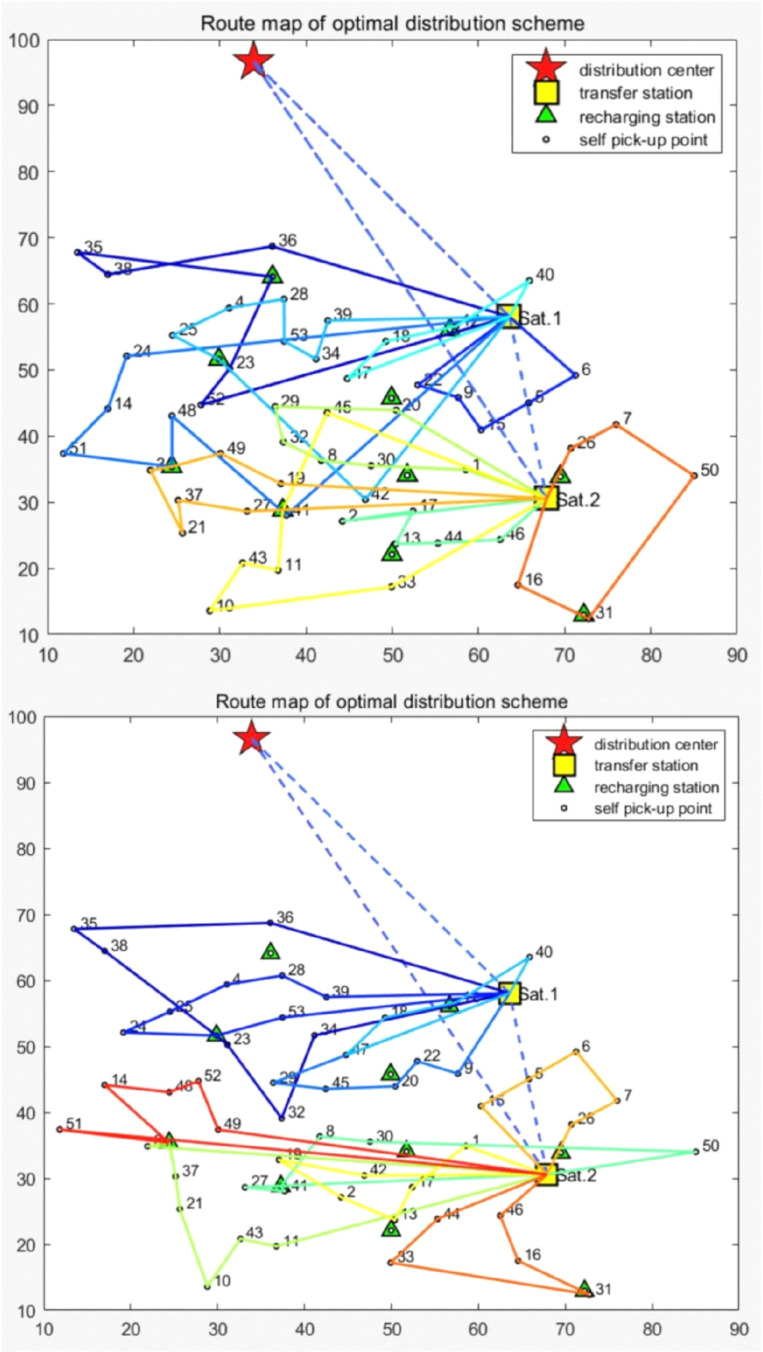
The optimal route of FR (above) and PR (below) scheme.

The routing method is utilized to calculate two distinct distribution strategies based on the case of company A’s self-established logistics network in Beijing. Each recharge scheme is executed five times. Table 5 presents the outcomes of the full/partial recharge scheme, with recharging station nodes highlighted in bold font. RUL represents the abbreviation for recharging the upper limit. The optimal outcome’s route map is illustrated in Fig 2. The analysis reveals that both the full and partial recharge schemes require a fleet of 3 vehicles in the first echelon and 10 vehicles in the second echelon to ensure efficient distribution. Additionally, enroute recharging is required for 5 EVs in the second echelon. In comparison, the full recharge strategy holds a slight advantage over the partial recharge strategy due to its lower overall cost. Furthermore, it is noteworthy that each station’s optimal solution under the partial recharge scheme indicates an approximate 100% recharge amount.

**Table 5:** The optimal result.

Charging type	initial node	routes	RUL	total cost
FR (full recharge)	distribution center	3->1->3		3715.804
		3->2->3		
		3->1->2->3		
	transfer station 1	64->36->38->35->**59**->23->52->64	100%	
		64->6->5->15->9->22->64		
		64->24->14->51->**55**->48->41->64	100%	
		64->39->34->53->28->4->25->**63**->42->64	100%	
		64->40->12->18->47->64		
	transfer station 2	65->46->44->13->17->2->65		
		65->1->30->8->32->29->20->65		
		65->33->10->43->11->**54**->45->65	100%	
		65->27->37->21->3->**55**->49->19->65	100%	
		65->26->7->50->31->16->65		
PR (partial recharge)	distribution center	3->2->3		3744.15
		3->2->1->3		
		3->1->3		
	transfer station 1	64->36->35->38->**63**->23->32->34->64	100%	
		64->39->28->4->25->24->**63**->53->64	90%	
		64->9->22->20->45->29->64		
		64->40->12->18->47->64		
	transfer station 2	65->50->30->8->**54**->41->27->65	100%	
		65->11->43->10->21->37->**55**->3->65	100%	
		65->1->17->13->2->19->42->65		
		65->26->7->6->5->15->65		
		65->46->16->31->33->44->65		
		65->49->52->48->14->**55**->51->65	100%	

In order to determine the optimal scheme for this specific case, the average costs of five runs in each scheme are compared and presented in Table 6. The limited literature on mixed fleet scenarios in a two-echelon cold chain network necessitates an examination of the effectiveness of the 2EVRPMF model through analysis of a case involving a diverse fuel vehicle fleet. In Table 6, ‘MF’ refers to the full recharge scheme for a mixed fleet, while ‘HF’ represents a fuel heterogeneous fleet.

**Table 6:** Average result of 5 runs with MF and HF.

		Fixed cost	Energy consumption cost	Deterioration cost	Carbon emission cost	Total cost	Distribution time
Echelon 1	MF	600.00	329.79	48.72	114.91	1101.44	4.96
	HF	600.00	329.79	42.91	114.91	1091.62	4.96
	Dev.	0%	0%	11.9%	0%	0%	0%
Echelon 2	MF	2000.00	437.67	127.70	78.42	2643.79	23.58
	HF	2000.00	757.15	107.15	254.27	3118.64	21.95
	Dev.	0%	-42.20%	19.18%	-69.16%	-15.23%	7.43%

Note: ‘dev’ denotes the percentage deviation of the solution obtained by MF from that obtained by HF scheme, with a negative ‘dev’ indicating superior performance of MF.

The results in echelon 1 demonstrate comparable performance between MF and HF, as both employ fuel vehicles at this tier. In echelon 2, the energy consumption cost of MF is significantly lower by 42.20% compared to HF due to the considerably higher unit price of fuel relative to electricity. Furthermore, the utilization of EVs in echelon 2 leads to a remarkable reduction of 69.16% in carbon emission costs. Although HF holds a slight advantage concerning deterioration costs and distribution time, there remains a noticeable disparity in total costs when compared with MF. Hence, it becomes apparent that this proposed model for 2EVRPMF exhibits superior performance, as it not only reduces overall expenses but also contributes towards sustainable development through decreased carbon emissions.

### 5.4. Sensitivity analysis

To establish an appropriate policy for the 2EVRPMF model, it is crucial to investigate the performance of each scheme under various scenarios. Considering the inherent connection between recharge schemes with EVs and recharging stations, factors such as battery capacity and recharging rate must also be taken into consideration. Consequently, a sensitivity analysis has been conducted specifically focusing on evaluating the effectiveness of FR and PR schemes within three instances (E-n51-k5-s6-12-32-37, E-n51-k5-s2-4-17-46 and E-n51-k5-s2-17) selected from benchmark instance Set 2c.

#### 5.4.1. Battery capacity.

The battery capacity range is defined as $B=\left[22.533.544.55\right]*D$, where *D* refers to the maximum distance between customers and their nearest recharging stations. B=2D serves as the lower limit for an EV to ensure it can complete a round trip from any customer to its closest recharging station. Additionally, B=5D is set as the upper limit for an EV to guarantee it can reach the nearest recharging station from any customer while maintaining a safe SoC (20%).

The influence of *B* is examined through the execution of 5 trials for 7 different capacities, incorporating 2 recharge schemes. The analysis reveals a significant decrease in the average total cost as the battery capacity increases. Specifically, there is a reduction of approximately 25% when the capacity is increased from 2D to 5D Regarding scheme performance, it becomes evident that the PR scheme does not consistently outperform the FR scheme. In fact, the FR scheme demonstrates superior performance when the capacity is below a certain threshold 2.5D as EVs tend to recharge at lower levels of capacity in order to efficiently fulfill their distribution tasks. However, the advantages of the PR scheme become apparent as battery capacity increases and restrictions are relaxed. The most noticeable disparity occurs around a battery capacity of 4D where an average deviation close to -3.5% can be observed between schemes. Nevertheless, with further increases in capacity, this deviation narrows.

#### 5.4.2. Recharging rate.

Nowadays, recharging stations are commonly categorized into AC and DC types. The AC station typically offers a charging rate of 7kWh/h, while the DC station provides rates ranging from 30 to 120kWh/h. Therefore, two recharge schemes were considered for discussing the charging rate. Results show that the total cost exhibits a significant decline of approximately 33.3% when the recharging station transitions from AC to DC. Subsequently, there is a relatively gradual decrease in the total cost within the range of recharging rates from 0.5kWh/min to 2kWh/min. Moreover, the average total cost consistently decreases as the recharging rate increases.

The PR scheme demonstrates significantly superior performance at the extremes of recharging rate, particularly at the highest or lowest levels, with the maximum deviation observed at a recharging rate of 0.12 kwh/min. When the rate is set to a moderate level, there is only a marginal deviation. Nevertheless, from an overall perspective, the PR scheme still surpasses other alternatives.

Therefore, it can be inferred that a larger battery capacity results in reduced overall costs. In terms of recharging rate, the DC recharging station is recommended due to its rapid charging speed and superior performance. To minimize overall expenses, technological advancements are necessary to enhance both battery capacity and recharging rate. When selecting recharge schemes, the PR scheme generally outperforms the FR scheme. However, in certain scenarios, the FR scheme may offer more advantages. This information can aid decision-making within the logistics industry regarding appropriate battery capacities, recharging rates, and recharge schemes.

### 5.5. Managerial insights

This paper presents a two-stage hybrid fleet routing model for perishable goods, to optimize fixed delivery costs, energy consumption costs and deterioration costs to the greatest extent possible. The study also takes into account the impact of both full and partial charging on the model’s outcomes. It demonstrates that utilizing fuel vehicles in suburban areas and electric vehicles in urban areas is not only cost-effective but also environmentally friendly. The number of vehicles required for different charging strategies is comparable, with only minor variations in overall cost performance observed. The capacity of charging stations and battery capacity of electric vehicles impose limitations on total costs. These findings can assist enterprises in effectively managing their fleets based on local charging station infrastructure and EVs technology, ultimately leading to long-term cost optimization.

## 6. Conclusions

The paper proposes an innovative two-echelon distribution scheme for cold chain logistics, aimed at promoting sustainable development in the industry. This scheme utilizes conventional fuel vehicles for first-echelon distribution and EVs for second-echelon distribution. A split delivery strategy is thoroughly investigated for the first echelon, along with two distinct recharge strategies for EVs in the second echelon. Additionally, a candidate matrix and corresponding pheromone matrix are constructed to determine an appropriate recharging limit - a concept initially introduced in the partial recharge strategy for simplifying selection processes. Finally, vehicle allocation and route selection methods are applied to solve this 2EVRPMF problem. The findings suggest that implementing this mixed fleet-based two-echelon vehicle routing optimization method can significantly enhance decision-making capabilities within the cold chain logistics industry:

1. The mixed fleet demonstrates superior performance compared to the heterogeneous fuel fleet, as it boasts a lower overall cost and effectively reduces carbon emissions by approximately 70%.
2. The battery capacity demonstrates a negative correlation with the total cost, resulting in a potential reduction of approximately 25% in the total cost when there is an increase in battery capacity from 2D to 5D
3. The recharging rate exhibits a negative correlation with the total cost. Transitioning from an AC to a DC recharging station can lead to a significant reduction in costs, approximately 33.3%. As the recharging rate of the DC station continues to increase, it will result in a decrease in the overall cost.
4. The PR scheme consistently outperforms the FR scheme. PR can achieve maximum savings of 3.5% when the battery size falls within the mid-range, and up to 4.5% when utilizing an AC recharging station. However, in specific scenarios with limited battery capacity, FR may present a more optimal solution.

In this paper, the investigation assumed that fuel and battery discharge are solely dependent on distance, in order to align with the company’s pursuit of interest and focus on the main issue. Moreover, it failed to consider customers’ time, which limits its applicability and leads to shortcomings in managing the entire logistics cycle. The future research should take into account the temporal constraints imposed by customers’ time windows, as this will impose more stringent limitations on the selection of two recharge strategies. Furthermore, a more precise investigation into the upper limit for recharging is warranted. Therefore, further discourse will concentrate on integrating time windows and refining the upper threshold for recharging.

## Supporting information

S1 Table Coordinates and demands of nodes in two-echelon distribution network. (DOCX)
