# Supplementary material for: Mixed fleet-based two-echelon vehicle routing optimization for cold chain logistics with diverse recharging strategies
Source: PLoS One. 2025 Feb 13;20(2):e0318765. doi: 10.1371/journal.pone.0318765 (PMC11825020; doi:10.1371/journal.pone.0318765)
Supplement: S1 Table — (DOCX) [file pone.0318765.s001.docx]

**S1 Table. Coordinates and demands of nodes in two-echelon distribution network.**

| No | X/km | Y/km | Demand/pcs |
| --- | --- | --- | --- |
| 1 | 113.10 | 13.80 | 189 |
| 2 | 114.95 | 15.55 | 72 |
| 3 | 101.75 | -54.60 | 159 |
| 4 | 34.60 | 39.80 | 78 |
| 5 | 159.95 | 74.40 | 67 |
| 6 | 169.75 | 9.45 | 168 |
| 7 | 174.75 | 65.65 | 111 |
| 8 | 103.90 | 13.40 | 93 |
| 9 | 34.90 | 24.30 | 77 |
| 10 | 172.90 | -2.00 | 108 |
| 11 | 150.15 | 66.00 | 95 |
| 12 | 160.45 | 19.60 | 60 |
| 13 | 192.70 | -28.25 | 113 |
| 14 | 153.95 | -9.30 | 152 |
| 15 | 130.85 | 10.15 | 131 |
| 16 | 41.90 | 55.35 | 115 |
| 17 | 144.15 | 25.75 | 149 |
| 18 | 199.85 | -15.60 | 138 |
| 19 | 112.75 | -23.25 | 82 |
| 20 | 124.20 | 53.40 | 93 |
| 21 | 39.75 | 50.15 | 96 |
| 22 | 181.70 | 6.35 | 102 |
| 23 | 187.20 | 91.10 | 134 |
| 24 | 45.05 | 55.50 | 154 |
| 25 | 176.40 | 76.15 | 199 |
| 26 | 62.35 | 42.55 | 190 |
| 27 | -6.40 | 24.70 | 125 |
| 28 | 138.50 | 29.40 | 136 |
| 29 | 166.25 | 1.75 | 186 |
| 30 | 39.05 | 38.45 | 73 |
| 31 | 74.00 | 33.55 | 56 |
| 32 | 132.20 | -10.05 | 135 |
| 33 | 114.55 | 11.65 | 98 |
| 34 | 117.55 | -3.55 | 61 |
| 35 | 49.70 | 52.35 | 67 |
| 36 | 114.05 | -21.10 | 71 |
| 37 | 135.00 | -13.45 | 59 |
| 38 | 117.70 | -30.10 | 198 |
| 39 | 126.35 | 33.90 | 191 |
| 40 | 100.10 | 26.60 | 57 |
| 41 | 176.25 | 30.05 | 69 |
| 42 | 157.30 | 11.00 | 54 |
| 43 | 114.40 | -6.65 | 86 |
| 44 | 121.85 | 51.90 | 165 |
| 45 | 161.95 | 49.20 | 84 |
| 46 | 150.65 | 22.10 | 160 |
| 47 | 164.75 | -4.25 | 67 |
| 48 | 176.40 | 6.15 | 78 |
| 49 | 62.85 | -28.60 | 107 |
| 50 | 182.70 | 30.75 | 52 |
| 51 | 103.40 | -9.00 | 89 |
| 52 | -6.90 | 22.45 | 126 |
| 53 | 181.00 | 52.55 | 196 |
| 54 | 64.85 | -35.25 | 168 |
| 55 | 145.30 | -20.80 | 87 |
| 56 | 126.35 | 2.35 | 170 |
| 57 | 182.40 | -2.25 | 180 |
| 58 | 88.60 | -17.15 | 91 |
| 59 | 129.50 | -19.65 | 56 |
| 60 | 146.50 | 50.50 | 95 |
| RC1 | 143.65 | 22.10 | 0 |
| RC2 | 149.30 | 27.00 | 0 |
| RC3 | 187.85 | 79.85 | 0 |
| RC4 | 132.50 | 45.55 | 0 |
| RC5 | 156.10 | -13.15 | 0 |
| RC6 | 69.65 | 29.95 | 0 |
| RC7 | 150.45 | 56.20 | 0 |
| RC8 | 133.35 | -4.75 | 0 |
| RC9 | 43.55 | 43.20 | 0 |
| RC10 | 109.55 | -13.15 | 0 |
| RC11 | 128.00 | 14.00 | 0 |
| RC12 | 175.05 | 27.35 | 0 |
| S1 | 77.80 | 67.85 | 0 |
| S2 | 165.45 | 49.20 | 0 |
| S3 | 205.65 | -25.60 | 0 |
| S4 | 134.35 | -63.85 | 0 |
| S5 | 62.50 | -52.25 | 0 |
| S6 | 104.40 | -4.20 | 0 |
| DC1 | 0.00 | 0.00 | 0 |

*Note: The ratio between the coordinate value and the actual distance (in kilometers) is 5:1.*
